# Glucose control during Ramadan fasting in a teenager with type 1 diabetes on MiniMed 670G hybrid closed-loop system

**DOI:** 10.1007/s00592-019-01414-6

**Published:** 2019-08-31

**Authors:** Goran Petrovski, Fawziya Al Khalaf, Judith Campbell, Khalid Hussain, Hannah Fisher, Fareeda Umer

**Affiliations:** Division of Endocrinology and Diabetes, Department of Pediatric Medicine, Sidra Medicine, HB 6E 219, Al Luqta Street, Education City North Campus, PO Box 26999, Doha, Qatar

## Introduction

Fasting during the month of Ramadan includes abstinence from drink and food from dawn till sunset and can be from several hours to more than 20 h per day. Prepubertal children and people with acute or chronic medical conditions are excused from fasting, which may aggravate their condition [1]. Children with type 1 diabetes (T1D) are considered a high-risk population, and it is recommended not to fast during Ramadan. However, a significant number of patients prefer to fast to respect their religion.

One of the treatment’s options for T1D is the MiniMed 670G hybrid closed-loop (HCL) system (Medtronic, USA), which uses an algorithm capable of automatically adjusting basal insulin delivery in response to glucose sensor readings transmitted to the insulin pump every 5 min.

We present the case with T1D where the glucose control was managed using the MiniMed 670G HCL system during the month of the Ramadan. To the best of our knowledge, this is the first patient to be reported on fasting during Ramadan and HCL system.

## Case presentation

A 13-year-old male patient with a 4-year history of T1D using MiniMed 670G HCL system was fasting for the first time around 14 h per day for the month of Ramadan. Patient had HbA1c levels between 8.2 and 11.8% (66–105 mmol/mol) with previous treatment (multiple daily injections with self-monitoring of blood glucose). MiniMed 670G HCL system was initiated 5 months before the study, and HbA1c of 6.6% (49 mmol/mol) and time in range (70–180 mg/dl; 3.9–10.0 mmol/l) of 76%) were achieved. The patient uploaded the HCL system on Carelink Personal Software on days 1, 14 and 30 during the Ramadan, and consultation was given by phone. Glucose and insulin metrics were analyzed 1 month before and during Ramadan period.

Patient broke the fast twice in the afternoon period during the first week of Ramadan due to a mild hypoglycemic event. We advised him to use temporary target for 2–4 h if glucose levels reached 80 mg/dl (4.4 mmol/l) to avoid further glucose decrease.

**Fig. 1:**
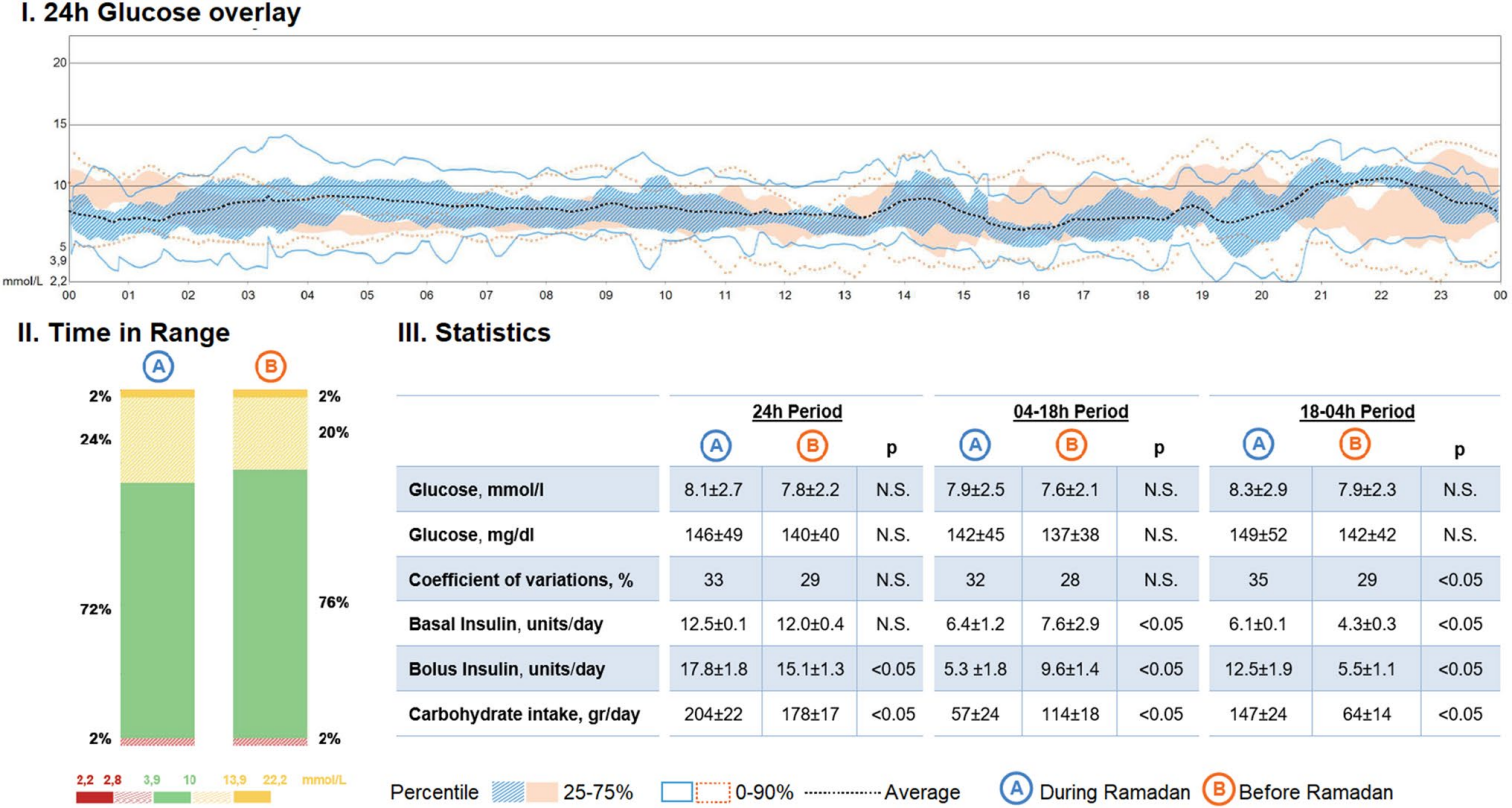
Glucose control during and before Ramadan. I. 24-h glucose overlay, percentile comparison of two periods; blue color, one month during Ramadan; orange color, one month before Ramadan. II. Time in range, glucose distribution comparison. III. Statistics, glucose and insulin metrics comparison. Glucose, basal insulin, bolus insulin and carbohydrates are expressed as mean plus SD for selected periods: 24 h, 04–18 h and 18–04 h. A-period, 1 month during Ramadan; B-period, 1 month before Ramadan (color figure online)

Despite correct carbohydrate counting, a slight increase in glucose values (18–00 h) was noted due to breaking the daily fast with eating at evening meal, iftar (as shown in Fig. 1). We recommended to increase the meal bolus by 10–20%, if the meal contained more than 100 g (e.g., to increase the bolus by 20% when 110 g of carbohydrates were eaten, 132 g of carbohydrates was entered into the bolus wizard calculator) and to split bolus insulin 40–50% before and 50–60% after the meal, as the “dual wave” and “square” boluses are disabled in MiniMed 670G HCL system.

Average auto-basal/basal insulin amount per day (as shown in Fig. 1) was significantly lower in the period 4 am–6 pm in fasting hours during Ramadan compared to the same period in the non-fasting hours before Ramadan (*p* < 0.05).

We did not find any significant difference in time spent in AM, AM exits, sensor wear and total daily insulin per day before and during Ramadan (as shown in Fig. 1). No diabetic ketoacidosis and severe hypoglycemia were detected during and before Ramadan.

## Discussion and conclusion

Despite certain limitations, we have provided a general overview on successful glucose control in child with T1D using the MiniMed 670G HCL system during the month of Ramadan.

Our case presented satisfactory glucose control without significant change in time in ranges, without influencing percentage of time spent with low glucose values 1 month during and 1 month before Ramadan. Like our findings, insulin pump can significantly reduce hypoglycemic events without increasing HbA1c values [2].

Insulin pumps and multiple daily injections are comparable in relation to glycemic control during Ramadan fasting in patients with T1D, where healthcare professionals are required to provide Ramadan-specific diabetes care. In our case, we did not provide specific diabetes care before Ramadan, which shows simple management of HCL system in T1D during Ramadan fasting.

Our case shows minimal adjustment of the HCL system before and during Ramadan with using a temporary target in the afternoon and increasing the meal bolus by 10–20% for huge meals in the evening, without any change in basal and bolus insulin settings. Our results differ to recently published studies on open-loop CSII, which have shown additional time in counseling on dietary modifications and therapeutic changes [3]. Automatically basal adjustments with MiniMed 670G are more practical during Ramadan compared to the clinical guidelines, where the reduction in basal rate by 5–50% for fasting days and weekly reassessment for further adjustments are recommended [4].

Like other authors, we did not find any diabetic ketoacidosis and severe hypoglycemia during the month of Ramadan [5].

HCL system with automatic adjustments of basal insulin delivery in response to CGM readings, temporary target feature, good collaboration between health providers and patient allows satisfactory glucose control during fasting of 14 h per day during the month of Ramadan, which we believe would not have been achieved using multiple daily insulin regimen or CSII. Further clinical trials should be performed to confirm our findings.

## References

[1] Abolaban H, Al-Moujahed A (2017). Muslim patients in Ramadan: a review for primary care physicians. Avicenna J Med.

[2] Elbarbary NS (2016). Effectiveness of the low-glucose suspend feature of insulin pump during fasting during Ramadan in type 1 diabetes mellitus. Diabetes Metab Res Rev.

[3] Afandi B, Kaplan W, Al Hassani N (2017). Correlation between pre-Ramadan glycemic control and subsequent glucose fluctuation during fasting in adolescents with type 1 diabetes. J Endocrinol Invest.

[4] Bajaj HS, Abouhassan T, Ahsan MR (2019). Diabetes Canada position statement for people with types 1 and 2 diabetes who fast during Ramadan. Can J Diabetes.

[5] Gad H, Al-Muhannadi H, Mussleman P (2019). Continuous subcutaneous insulin infusion versus multiple daily insulin injections in patients with type 1 diabetes mellitus who fast during Ramadan: a systematic review and meta-analysis. Diabetes Res Clin Pract.

